# Amniotic Fluid Stem Cells Prevent Follicle Atresia and Rescue Fertility of Mice with Premature Ovarian Failure Induced by Chemotherapy

**DOI:** 10.1371/journal.pone.0106538

**Published:** 2014-09-08

**Authors:** Guan-Yu Xiao, I-Hsuan Liu, Chun-Chun Cheng, Chia-Chun Chang, Yen-Hua Lee, Winston Teng-Kuei Cheng, Shinn-Chih Wu

**Affiliations:** 1 Institute of Biotechnology, National Taiwan University, Taipei, Taiwan; 2 Department of Animal Science and Technology, National Taiwan University, Taipei, Taiwan; 3 Research Center for Developmental Biology and Regenerative Medicine, National Taiwan University, Taipei, Taiwan; 4 Department of Animal Science and Biotechnology, Tunghai University, Taichung, Taiwan; University of Connecticut, United States Of America

## Abstract

Chemotherapy used to treat cancer may cause irreversible premature ovarian failure (POF). Of late, amniotic fluid stem cells (AFSCs) provide a novel source for regenerative medicine because of their primitive stage, low immunogenicity, and easy accessibility. In this study, we isolated AFSCs from transgenic mice that ubiquitously express enhanced green fluorescence protein (EGFP). These AFSCs exhibited morphologies, immunophenotypes, and mesoderm trilineage differentiation potentials similar to mesenchymal stem cells (MSCs). Further, AFSCs proliferated faster than MSCs and expressed OCT4, a marker for pluripotency. To investigate their potential in recovering fertility in POF model, AFSCs were transplanted into the ovaries of mice with POF six weeks post induction using chemotherapeutic drugs, busulfan and cyclophosphamide. AFSCs could rescue the reproductive ability of mice with POF by preventing follicle atresia and sustaining the healthy follicles. Notably, the transplanted AFSCs did not differentiate into granulosa and germline cells *in vivo*. After one month, the decreased numbers of transplanted AFSCs accompanied with the reduced beneficial effects indicated that the therapeutic efficacy were directly from AFSCs. These findings demonstrated the therapeutic effects of AFSCs and suggested the promise of AFSCs for treating infertility and POF caused by chemotherapy.

## Introduction

Chemotherapy (CTx) is commonly used for treating various malignancies and can improve the survival rate for cancer patients [1]. A potential adverse effect of chemotherapy is to decrease the number of ovarian follicles, disrupt the menstrual cycle, induce infertility, and cause irreversible premature ovarian failure (POF) [2]–[4]. In addition, early onset of menopause can result in the reduction in the quality of life. POF is associated with risks of cardiovascular disease [5], osteoporosis [6], and psychiatric diseases such as depression [7]. A recent study showed that bone marrow transplantation can rescue the fertility of mice with CTx [8]. Subsequently, more studies proved that transplantation of mesenchymal stem cells (MSCs) could restore partial ovarian function in a mouse model of CTx-induced POF [9], [10]. These findings suggested the potential of MSCs to treat ovarian failure in humans, but its cellular and molecular mechanisms are still unknown. Furthermore, the proliferative capacity of MSCs *in vitro* is limited, and its acquisition is highly invasive [11]; therefore, an alternative cell source in clinical POF applications may be preferred.

To address this issue, we reasoned that amniotic fluid stem cells (AFSCs) may serve as an alternative option because they are readily harvested from amniotic fluid, undergo rapid self-renewal, maintain a normal karyotype in long-term culture [12], and tally with all the properties defined in the minimum criteria for MSCs [13]–[16]. Compared with other MSCs, AFSCs are easy to harvest, have a shorter doubling time and express the embryonic stem cells (ESCs) marker OCT4 [17], whereas unlike ESCs and induced pluripotent stem cells, they do not develop into teratomas *in vivo* [18]. Further, evidence indicated that transplanted allogeneic AFSCs have low immunogenicity and can escape immune rejection [19]. The potential advantages of AFSCs for treating POF are further suggested by their use in the studies of regenerative medicine to repair damaged tissues or organs, such as muscle [14], kidney [20], lung [21], or heart [22].

In this study, we evaluated the efficacy of AFSCs in a mouse model of POF and investigate the potential mechanism of this therapeutic effect. We established and characterized AFSCs from transgenic mice ubiquitously expressing enhanced green fluorescence protein (EGFP), which enables us to trace the fate of AFSCs after transplantation. To evaluate whether AFSCs can rescue the female fertility, we transplanted AFSCs expressing EGFP (EGFP-AFSCs) into the ovaries of mice with POF, assessed ovarian function and traced the fate of transplanted AFSCs within the ovaries of recipients.

## Materials and Methods

### Animals and Ethics Statement

ICR mice aged 4–6 weeks were purchased from the Laboratory Animal Center of Medical College of National Taiwan University (Taipei, Taiwan). EGFP transgenic mice that ubiquitously express EGFP were as previously described [23]. In brief, EGFP-expressing transgenic mouse lines were created by pronuclear microinjection of ICR strain zygotes with a construct (pCX-EGFP) that expresses EGFP under control of the *β-actin* promoter [24]–[26]. Mice were housed under a 14–10 h light–dark cycle at 25°C±2°C with food and water provided *ad libitum*. The Institutional Animal Care and Use Committee of National Taiwan University approved the experimental protocol (NTU-102-EL-10).

### Isolation and Culture of EGFP-AFSCs

Mice were euthanized by sequential CO_2_ asphyxiation and cervical dislocation at day 11.5 of pregnancy. The uterus was isolated and 26-gauge needles were used to collect amniotic fluid from each EGFP-expressing conceptus. Amniotic fluid was added to the wells of a 24-well cell culture dish (#92024, TPP, Trasadingen, Switzerland) containing 1 mL of Minimum Essential Medium (MEM)-α (#M0894, Sigma-Aldrich, St. Louis, MO) supplemented with 3.7 mg/mL NaHCO_3_ (#S5761, Sigma-Aldrich), 10% (v/v) fetal bovine serum (FBS) (#SH30070-03, GE Healthcare Life Sciences, South Logan, Utah), 100 IU/mL penicillin, and 100 mg/mL streptomycin (#15140, Gibco, Grand Island, NY). Cells were incubated at 37°C in a humidified atmosphere of 5% CO_2_. Every three days, 0.5 mL of non-adherent cells were removed and replaced with 0.5 mL of fresh medium. Cells were passaged after reaching 70%–80% confluence after incubation at 37°C with 0.25% (w/v) trypsin/ethylenediaminetetraacetic acid (EDTA) (#2500056, Gibco) for 5 min.

### Flow Cytometry Analysis

To assess the immunophenotypes of cultured AFSCs, we performed a flow cytometric assay. Cells were reacted in the dark for 30 min at 4°C according the manufactures' protocols with the following phycoerythrin (PE)-conjugated monoclonal antibodies: CD11b (#12-0112), CD29 (#12-0291), CD31 (#12-1051), CD34 (#12-1051), CD44 (#12-0441), CD45 (#12-0451), MHC-I (#12-5998), MHC-II (#12-5321) and Sca-1 (#12-5981). A FACSCalibur (BD Biosciences, Franklin Lakes, NJ) was used to analyze 1×10^4^ cells, and the data were processed using the FCS Express software (Version 4.0; Denovo Software, Los Angeles, CA). All antibodies included isotype-matched control antibodies were purchased from eBioscience (San Diego, CA).

### Mesoderm Trilineage Differentiation of EGFP-AFSCs

In order to investigate whether the cultured EGFP-AFSCs have the mesoderm trilineage differentiation potentials as MSCs, the differentiation ability test is based on the induction system with modifications. To induce adipogenesis, near-confluent monolayers of EGFP-AFSCs were cultured in MEM-α supplemented with 10% FBS, 10 µg/mL insulin (#I6634), 1 µM dexamethasone (#D4902), 0.5 mM isobutyl-methylxanthine (#I5879), and 100 µM indomethacin (#I7378) for 14 days with medium changes every three days. Cells were fixed with 4% paraformaldehyde (PFA) (#P6148) at room temperature for 10 min, and lipid droplets were stained using Oil Red O (#O9755) [27].

To induce osteogenesis, confluent cells were incubated in MEM-α supplemented with 10% FBS, 0.1 µM dexamethasone, 10 mM β-glycerolphosphate (#G9891) and 50 µM ascorbic acid (#A8960) for 21 days with medium changes every three days. Bone matrix mineralization was evaluated using Alizarin Red S staining (ARS) (#A5533) [27].

A pellet culture system was used to induce chondrogenesis with MEM-α supplemented with 1% FBS, 6.25 µg/mL insulin, 50 µM ascorbic acid and 10 ng/mL transforming growth factor (TGF)-β1 (#240-B-002, R&D Systems, Minneapolis, MN) with twice weekly medium changes. Proteoglycan production was determined using toluidine blue O (#T3260) staining [28]. All chemicals were purchased from Sigma-Aldrich unless otherwise noted.

### Isolation and Culture of Bone Marrow MSCs

Bone marrow MSCs (BMMSCs) were isolated using a published method [29]. Femurs and tibias were isolated from euthanized ICR mice, and scissors were used to remove epiphyses. The marrow cells were harvested using 23- and 26-gauge hypodermic needles from the diaphysis of femurs and tibias, respectively, followed by flushing with MEM-α supplemented with 3.7 mg/mL NaHCO_3_, 10% FBS, 100 IU/mL penicillin and 100 mg/mL streptomycin. Cells (2×10^5^ cells/cm^2^) were added to 60-cm^2^ culture dishes and incubated as described above. Non-adherent cells were removed by changing the medium every three days. Cells (70% confluent) were removed from the dishes by incubation at 37°C with 0.25% trypsin/EDTA for 5 min and were purified using transient lower-density plastic adherence [23].

### Cell Proliferation

To assess the proliferative ability of EGFP-AFSCs, we estimated the population doubling time (PDT) according to the equation: TD = t_p_ log2/(logNt−logNo), where Nt and No are the numbers of cells harvested and inoculated, respectively, and t is the time of culture (in hours). Cell viability was determined using the trypan blue (#15250, Gibco) exclusion test, and the cells were counted using a hemocytometer [30].

### Immunocytochemistry

Cells were fixed with 4% (w/v) PFA for 10 min at room temperature and permeabilized using 0.25% Triton X-100 (v/v) (#0694, Amresco, Solon, OH) for 10 min. To reduce nonspecific antibody-binding, the cells were treated with 0.1% (v/v) Tween-20 (#X251-07, Sigma-Aldrich) in phosphate buffered saline (PBS) (#0780, Amresco) (PBST) containing 3% (w/v) bovine serum albumin (BSA) (#0332, Amresco) for 30 min and then incubated with a rabbit anti-OCT4 antibody (1∶200; #SC-9081, Santa Cruz, Dallas, TX) at 4°C overnight. Immune complexes were detected using a goat anti-rabbit IgG secondary antibody labeled with Alex Fluor 594 (1∶300; #A11012, Invitrogen, Carlsbad, CA). Nuclei were stained with 0.1 mg/mL 4′,6-diamidino-2-phenylindole (DAPI) (1∶1000; #40043, Biotium, Hayward, CA) for 3 min. The cells were observed using a fluorescence microscope (DMIRB; Leica, Wetzlar, Germany).

### Mouse Model of CTx-Induced POF

Six-week-old ICR female mice were administered busulfan (20 mg/kg) (#B2635) and cyclophosphamide (200 mg/kg) (#C0768) dissolved in dimethyl sulfoxide (DMSO) (#D2650) or DMSO only using a single intraperitoneal injection. All chemicals were purchased from Sigma-Aldrich. Mice were observed for 6 weeks. Mice treated with busulfan/cyclophosphamide or DMSO only are designated CTx- and non-CTx-mice, respectively.

### Cell Transplantation

After six weeks of the POF mouse model established successfully, EGFP-AFSCs were transplanted into the ovaries of CTx-mice using a modification of a published study [31]. In brief, a suspension of 5×10^5^ EGFP-AFSCs in 5 µL of sterilized PBS was injected through a glass pipette (100-µm tip) into each ovary of mice anesthetized with inhaled isoflurane (#B506, Abbott, Chicago, IL). CTx-mice with EGFP-AFSCs transplantation or PBS injection only are designated CTx-AFSCs- and CTx-saline-mice, respectively.

### Ovarian Morphology and Follicle Counts

The number of follicles in each ovary was estimated according to a modified published protocol [32]. Ovaries were fixed with 4% PFA at 4°C overnight, embedded in paraffin, serially sectioned (8 µm), and stained with hematoxylin-eosin. The number of primordial, primary, secondary, antral, or atretic follicles in every randomly selected tenth section was recorded. The different categories of follicles were determined as described previously [33]. Only follicles containing an oocyte with a clearly visible nucleus were scored.

### Vaginal Cytology

To validate the reproduction function, the number of estrous cycles will be analyzed by vaginal smear assay every day after one week post AFSCs transplantation and for 14 consecutive days of test. Vaginal cells were collected using a sterile cotton swab, smeared on glass microscope slides, and stained with 0.5% (w/v) methylene blue (#M9140, Sigma-Aldrich), and the cells were classified using a standard criteria [34]. An estrus cycle was defined as the period between two successive proestrus phases (proestrus  =  day 1).

### Mating Trial

Mating trial was initiated after one week post AFSCs transplantation and continued for 24 days. One fertile male were housed with one female mouse from each experimental group. The presence of a copulatory plug was defined as successful mating. Males were randomly rotated among cages after each pregnancy, and the number of offspring per litter was recorded.

### Immunohistochemistry

In brief, ovaries were fixed with 4% PFA at 4°C overnight, incubated in 30% sucrose (#S0389, Sigma-Aldrich) at 4°C for three days, frozen, sectioned, incubated with PBS containing 0.25% Triton X-100 for 15 min, and blocked with PBST containing 3% BSA for 30 min. Sections were incubated with a rabbit anti-VASA (DDX4) antibody (1∶200; #ab13840, Abcam, Cambridge, UK) for 1 h at room temperature, rinsed with PBS, and incubated with a goat anti-rabbit IgG secondary antibody labeled with Alex Fluor 594 (1∶300). Nuclei were stained with DAPI for 3 min. All sections were mounted with fluoroshield mounting medium (#ab104135, Abcam) on glass slides and observed using a confocal microscope (Leica TCS SP5, Wetzlar, Germany).

### Reverse Transcription-Polymerase Chain Reaction (RT-PCR) Assay

The total RNA was extracted from ovarian tissue using TRIzol reagent (#10296-010, Invitrogen). Reverse transcription reactions were performed using the SuperScript First-Strand Synthesis System (#18064-014, Invitrogen) with 1.6 µg total RNA. PCR was performed with Taq polymerase (#11615-010, Invitrogen). The preceding reactions were performed according to the manufacturers' instructions. PCR reactions were performed as follows: initial denaturation at 94°C for 5 min, 35 cycles of denaturation at 94°C for 30 s, annealing at 55°C for 30 s, elongation at 68°C for 30 s, and final incubation at 72°C for 10 min. The sequences of primers were as follows: forward 5′-AGAGGGAAATCGTGCGTGAC-3′, reverse 5′-CAAGAAGGAAGGCTGGAAAA-3′ for *Actb* (188 bp); forward 5′- TATATCATGGCCGACAAGCA-3′, reverse 5′- GAACTCCAGCAGGACCATGT-3′ for *egfp* (219 bp). The expression of *β-actin* was used as the internal control.

### Statistical Analysis

Three biological replicates were performed, and all values represent the mean ± standard error of the mean (S.E.M). An unpaired Student *t*-test and Tukey's multiple comparison test were used to compare the data for two groups and three groups, respectively. *P*<0.05 was defined as a statistically significant difference. Log-rank (Mantel-Cox) test was used to compare the percentage of successful mating for three groups and the *P*<0.01 was defined as a statistically significant difference.

## Results

### Characterization of EGFP-AFSCs

**Figure 1:**
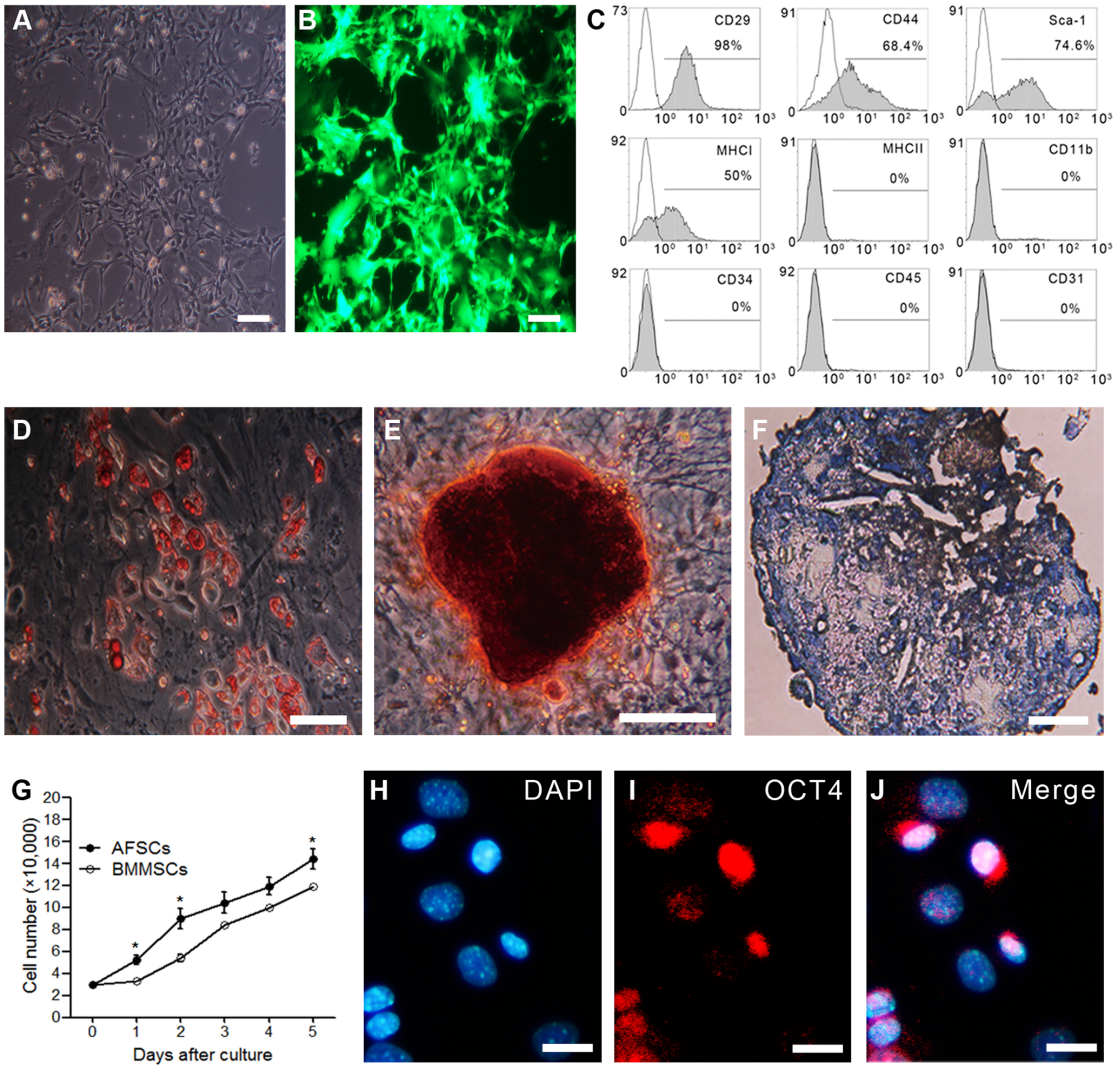
Characterization of EGFP-AFSCs in culture. (A) Amniotic fluid stem cells (AFSCs) (third passage) exhibit fibroblast-like morphology and adhere to the surface of plastic cell culture dishes. (B) Harvested AFSCs express enhanced green fluorescence protein (EGFP). (C) Immunophenotypes of AFSCs at their third passage expressed markers of mesenchymal stem cells (MSCs) but not those of hematopoietic and endothelial cells. The black histograms indicate the respective isotype control. (D) Oil Red O staining after 14 days of culture in adipogenic medium showed adipogenic differentiation potential of AFSCs. (E) Alizarin Red S (ARS) staining after 21 days of culture in osteogenic medium showed osteogenic differentiation potential of AFSCs. (F) Toluidine blue O staining after 21 days culture in induction chrondrogenic medium showed chondrogenic differentiation potential of AFSCs. Scale bars = 100 µm. (G) Proliferation ability of EGFP-AFSCs (third passage) was better than that of bone marrow MSCs (BMMSCs) (third passage) (n = 3). Data represent the mean ± standard error of mean (S.E.M.) *P<0.05. (H–J) Immunocytochemistry analysis revealed the OCT4 expression (red in I and J) by EGFP-AFSCs with nuclear counterstaining by 4′,6-diamidino-2-phenylindole (DAPI) (blue in H, J and K). Scale bar = 25 µm.

Most of the EGFP-AFSCs (Figure 1A) strongly expressed EGFP (Figure 1B), and flow cytometric analysis showed that cultured EGFP-AFSCs expressed CD29, CD44, Sca-1, and MHC-I, while did not express MHC-II, hematopoietic lineage markers (CD11b, CD34, CD45) or an endothelial cell marker (CD31) (Figure 1C). Culturing in adipogenic medium induced EGFP-AFSCs to form intracellular lipid droplets (Oil Red O-positive) (Figure 1D). Culturing in osteogenic medium induced EGFP-AFSCs to form calcium deposits and mature nodules structures, which were stained by ARS (Figure 1E). After 21 days of incubation in chondrogenic medium, EGFP-AFSCs stained with toluidine blue exhibited a blue-purple matrix (Figure 1F). These findings showed that AFSCs displayed morphology, immunophenotypes and mesoderm trilineage differentiation capacity similar to MSCs.

To further evaluate the proliferative ability of AFSCs, we performed growth curve and PDT assay compared to BMMSCs. We found that AFSCs showed higher cell population growth with shorter PDTs than BMMSCs (31.22±1.10 h and 34.41±0.73 in AFSCs and BMMSCs, respectively) (Figure 1G). In accordance with previous studies, we confirmed that most of cultured AFSCs expressed OCT4 in the nucleus by immunocytochemistry staining (Figure 1H-J). These findings illustrated that the AFSCs we established exhibited higher proliferation ability than BMMSCs and expressed OCT4.

### Effect of CTx on Ovarian Function and Induced POF

**Figure 2:**
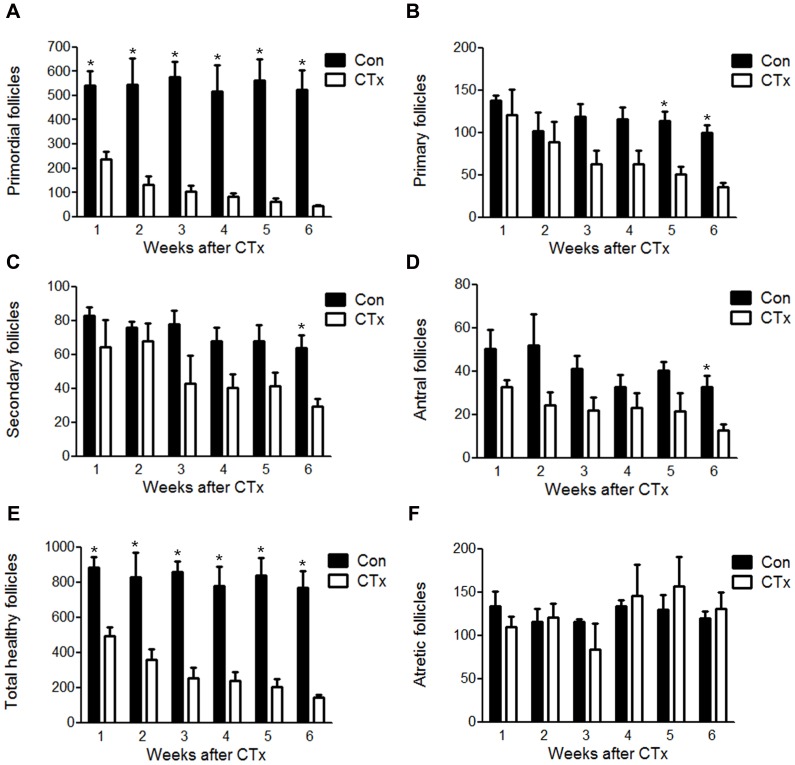
Quantitative analysis of ovarian follicles of mice with CTx. (A) Primordial, (B) primary, (C) secondary, (D) antral, (E) total healthy, and (F) atretic follicles were counted in the ovaries of mice with or without CTx. Con  =  Non-CTx-mice. CTx  =  CTx-mice. Data represent the mean ± standard error of mean (S.E.M.) of three experiments. *P<0.05.

The ovaries of CTx-mice were atrophic compared with those of non-CTx-mice and contained fewer developing follicles at the sixth week post CTx (Figure S1). Further, primordial follicles were significantly fewer in the ovaries of CTx-mice and almost disappeared after six weeks post CTx (Figure 2A). The numbers of developing and total healthy follicles were also significantly reduced in the ovaries of CTx-mice, particularly after six weeks (Figure 2B-E). In contrast, the numbers of atretic follicles were not significantly different between the ovaries of CTx- and non-CTx-mice (Figure 2F).

### AFSCs Prevent Follicular Atresia and Sustained the Development of Follicles in the Ovaries of CTx-mice

**Figure 3:**
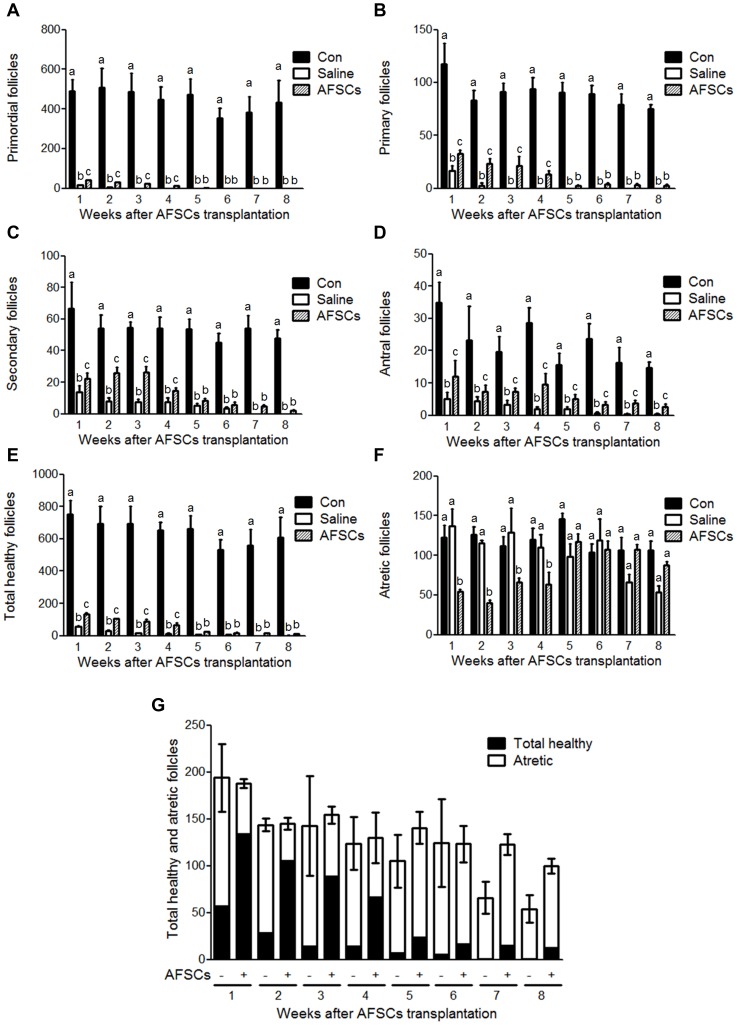
Quantitative analysis of ovarian follicles of CTx-mice transplanted with EGFP-AFSCs. (A–F) (A) Primordial, (B) primary, (C) secondary, (D) antral, (E) total healthy, and (F) atretic follicles were counted in the mice ovaries. (G) The transplantation of EGFP-AFSCs did not alter the number of the sum of total healthy and atretic follicles in the ovaries of CTx-mice. Con  =  Non-CTx-mice. Saline  =  CTx-saline-mice. AFSCs  =  CTx-AFSCs-mice. Data represent the mean ± standard error of mean (S.E.M.) of three experiments. Lower case letters (a, b, and c) represent significant differences (*P*<0.05) among groups.

To assess the therapeutic effect of AFSCs, we transplanted EGFP-AFSCs directly into bilateral ovaries of mice with POF six weeks after CTx. Although there were few primordial follicles in the ovaries of CTx-saline- and CTx-AFSCs-mice, during the course of the experiment, the numbers of primordial follicles were significantly higher in CTx-AFSCs-mice during the first four weeks post-transplantation (wpt) (Figure 3A). The results were similar for the numbers of primary and secondary follicles among the three groups (Figure 3B and C). The number of antral follicles of CTx-AFSCs-mice was significantly higher compared with that of CTx-saline-mice, whereas lower compared with that of non-CTx-mice at all times (Figure 3D). The number of total healthy follicles in CTx-AFSCs-mice was higher compared with CTx-saline-mice within 4 wpt and was significantly lower compared with that of non-CTx-mice at all times (Figure 3E). Injection of EGFP-AFSCs significantly decreased the number of atretic follicles up to 4 wpt. In contrast, there was no significant difference between non-CTx- and CTx-saline-mice at all times (Figure 3F). There was no significant difference between the sum of total healthy and atretic follicles of CTx-AFSCs- and CTx-saline-mice (Figure 3G). These results indicated that AFSCs could transiently prevent follicular atresia and sustain the follicular development in the ovaries of mice with POF.

### AFSCs Restore the Fertility of Mice with POF

**Figure 4:**
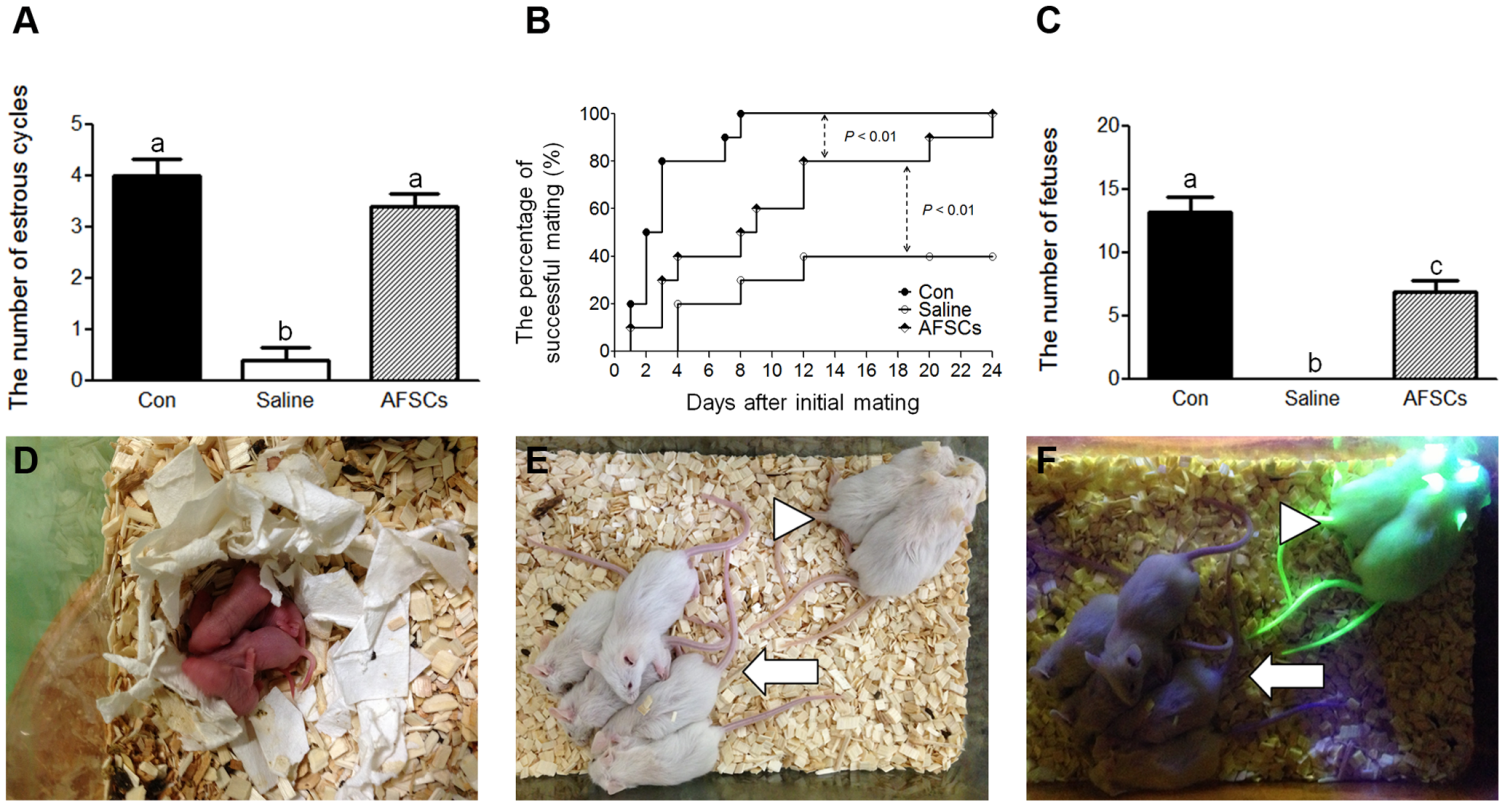
Reproductive function was improved in CTx-mice transplanted with EGFP-AFSCs. (A) The number of estrous cycles in two weeks was evaluated in the mice. Con  =  Non-CTx-mice (n = 5). Saline  =  CTx-saline-mice. (n = 5). AFSCs  =  CTx-AFSCs-mice. (n = 5). Data represent the mean ± S.E.M. Lower case letters (a, b, and c) represent significant differences (*P*<0.05) among groups. (B) The successful mating rate was assessed in a 24-day period. The presence of a copulatory plug indicated successful mating. Con  =  Non-CTx-mice. (n = 10). Saline  =  CTx-saline-mice. (n = 10). AFSCs  =  CTx-AFSCs-mice. (n = 10). Log-rank (Mantel-Cox) test was used to compare the data among groups. *P*<0.01 represents significant differences between groups. (C) The litter numbers were counted. Con  =  Non-CTx-mice (n = 10). Saline  =  CTx-saline-mice. (n = 4). AFSCs  =  CTx-AFSCs-mice (n = 10). Data represent the mean ± S.E.M. Lower case letters (a, b, and c) represent significant differences (*P*<0.05) among groups. (D–F) The F1 litters showed normal gross appearance with no progeny bearing EGFP (arrow) as compared to the EGFP transgenic mice (arrowhead).

The number of estrous cycles of CTx-AFSCs-mice (3.4±0.22) was significantly higher compared with that of CTx-mice (0.4±0.22) but not significantly different compared with that of non-CTx-mice (4±0.28) (Figure 4A). Although the CTx-AFSCs-mice reached 100% successful mating rate significantly slower than non-CTx control group (*P*<0.01), the CTx-saline-mice reached only 40% successful mating in the 24-day test period and was significantly worse than CTx-AFSCs-mice (*P*<0.01) (Figure 4B). Moreover, consistent with the numbers of antral follicles, the average number of litters produced by CTx-AFSCs-mice (6.9±0.85) was significantly higher compared with those of CTx-mice (0) and lower compared with those of non-CTx-mice (13.2±1.09) (Figure 4C).

### EGFP-AFSCs do not Contribute to the Germline in Ovaries of CTx-Mice

**Figure 5:**
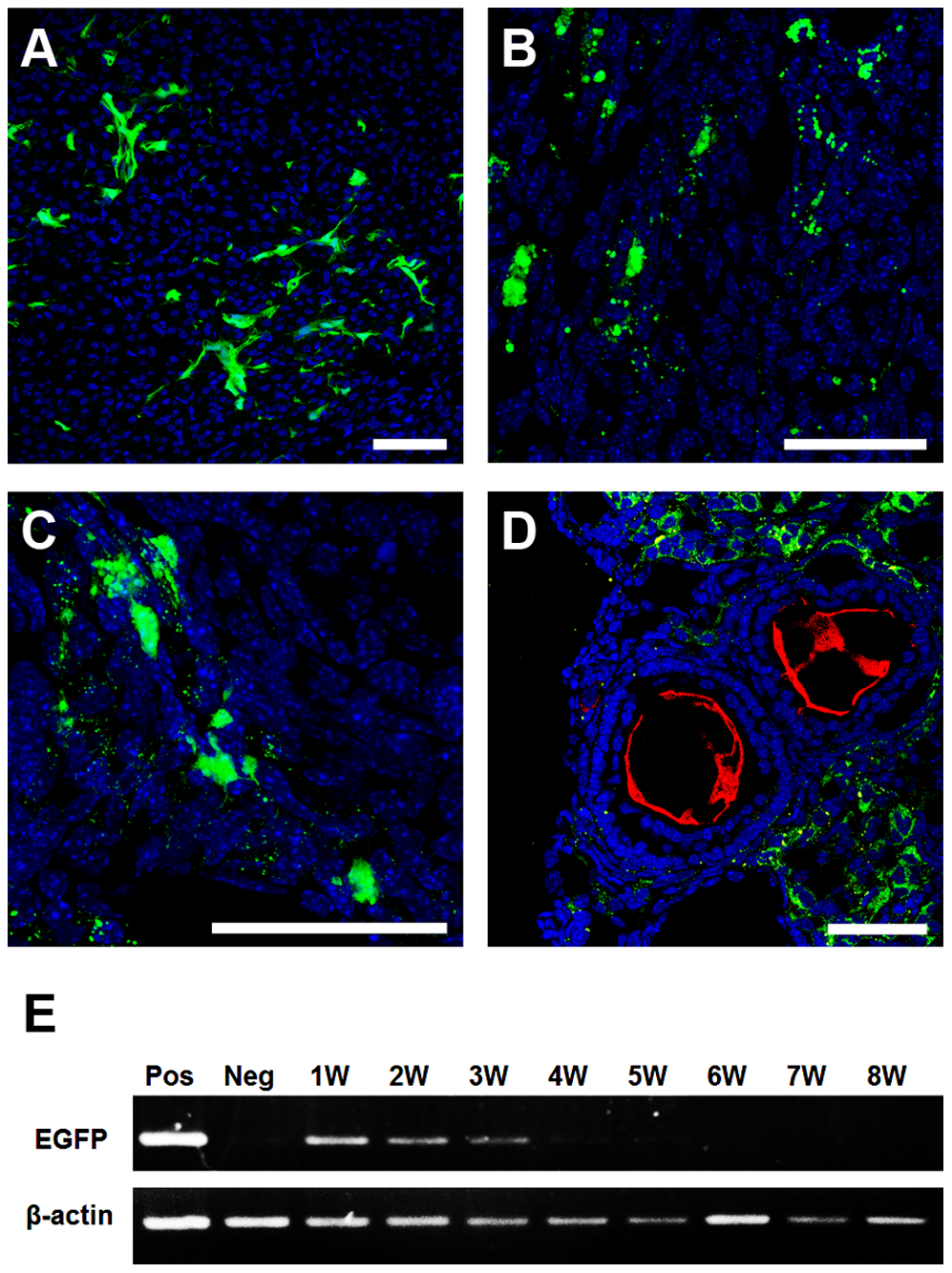
Immunohistochemical analyses of ovaries of CTx-mice transplanted with EGFP-AFSCs. (A–C) Transplanted EGFP-AFSCs (green) can be observed in the ovaries after one (A), two (B), and three (C) weeks of transplantation. DAPI (blue) was used to stain nuclei. Scale bar = 50 µm. (D) Analysis of VASA (red) and EGFP (green) expression showed that there is no co-localization in recipient ovaries at 4 weeks post-transplantation. Scale bar = 50 µm. (E) Reverse transcription polymerase chain reaction (RT-PCR) analysis of recipient ovaries at different times after EGFP-AFSCs transplantation. Ovarian tissues harvested from an EGFP mouse (Pos) and a CTx-saline-mouse (Neg) was used as a positive and negative control respectively. *β-actin* mRNA served as an internal control. W  =  Weeks.

The progeny of CTx-AFSCs-mice appeared normal (Figure 4D and E), and none expressed EGFP (Figure 4F), indicating that offspring were derived from the recipient's germline (n = 69). Therefore, we tracked the fate of the transplanted EGFP-AFSCs within the ovaries of recipients. EGFP-AFSCs were detected in ovarian tissue at 1 wpt and persisted for at least four weeks (Figure 5A-D). Neither the germ cell marker VASA [35] (Figure 5D) nor the granulosa cell marker follicle stimulating hormone receptor (FSHR) [36] (data not shown) was detectably expressed by EGFP-AFSCs. Moreover, the level of the mRNA encoding EGFP in the ovaries of CTx-AFSCs-mice decreased gradually after transplantation, and little was detected after 5 wpt (Figure 5E).

## Discussion

Cancer CTx using busulfan/cyclophosphamide are designed to destroy highly proliferating cells by inducing cross-linking of DNA [37], which affect mitochondria and result in the activation of apoptotic pathway [38]. Accordingly, it is widely believed that chemotherapeutic drugs may massively eliminate granulosa cells [39], which are required for oocyte survival and follicle development [40]. The ensuing damage to growing follicles by CTx increases recruitment of primordial follicles and, in turn, the number of all types of follicles reduced [41]. Consistent with this hypothesis and previous studies [42], [43], our results showed that CTx treatment significantly reduced various types of follicles and number of total healthy follicles (Figure 2A-E). Nonetheless, although CTx largely diminished number of total healthy follicles, the number of atretic follicles was not significantly different at any point during the 6 weeks observation (Figure 2F). According to the previous studies, chemotherapeutic drugs significantly increased follicle atresia predominantly within 72 h after the treatment and the number of atretic follicle quickly returned to the basal level within one week after the treatment. Subsequently, the degenerative follicles were removed from the ovary within a 3-day period [32], [44]. This should be the reason of the discordance in the observed numbers of follicle loss versus atresia. Consequently, the exhaustion of primordial follicles and continued follicular atresia severely impaired the ovarian function and resulted in POF after six weeks of CTx.

In this study, we demonstrated that AFSCs increased healthy ovarian follicles and restored fertility in a mouse model system of POF induced by CTx. In principle, AFSCs may sustain the number of healthy ovarian follicles in POF mice either by promoting *de novo* folliculogenesis or by inhibiting follicular atresia or both of these two mechanisms. Folliculogenesis is the process that the primitive oocytes, which originated from primordial germ cells [45], are then surrounded by pregranulosa cells in fetal ovary to form primordial follicles during embryogenesis [46]. Previous study reported that AFSCs expressed oocyte marker in vitro [47], and therefore it might be possible that the transplanted AFSCs have the potential to differentiate into primitive oocyte and participate in the following folliculogenesis within ovaries of POF mice. However, we found no progeny of CTx-AFSCs-mice were derived from the EGFP-AFSCs and none expressed EGFP. Moreover, transplanted EGFP-AFSCs were distributed within the ovarian interstitium rather than follicles and express neither a marker of differentiated germ cells (Figure 5D) nor of granulosa cells (data not shown). These results indicated that the transplanted AFSCs *per se* did not participate in the folliculogenesis.

We further showed a significant decrease in the number of atretic follicles in the ovaries of CTx-AFSCs-mice that persisted for four weeks (Figure 3F), which is in correlation with the existence period of EGFP-AFSCs (Figure 5E). Notably, the numbers of total healthy and atretic follicles were not significantly different between CTx-AFSCs-mice and mice that were not engrafted (Figure 3G). These data strongly suggested that AFSCs exerted therapeutic effect on increasing the number of healthy ovarian follicles by preventing follicular atresia.

According to previous research, follicular atresia is predominantly mediated by the apoptosis of follicular cells, especially granulosa cells which are necessary for follicular development [48]–[50]. Nonetheless, we found no evidence that transplanted AFSCs directly differentiated into granulosa cells to replace damaged cells within ovary of POF mice. It has been hypothesized that the restorative effects of MSCs transplantation on impaired ovarian function resulted from secretion of trophic factors, which are beneficial to follicle growth [9], [10]. Previous studies reported that AFSCs can secrete TGF-β [51], vascular endothelial growth factor [52], and glia cell-derived neurotrophic factor [53], which are required for follicular development [54], [55], and can inhibit follicular cell apoptosis [56] and follicle atresia [57]. Taken together, we infer that the favorable effects of AFSCs on CTx-damaged ovary is derived from the paracrine secretion of certain beneficial factors, which support ovarian follicle survival. Since ovarian granulosa cells are essential to sustain follicle survival, it is likely that the interaction between AFSCs and granulosa cells plays a key role in this favorable effect. To address this issue, establishment of the granulosa cells and AFSCs *in vitro* co-culture system for screening the candidate factors shall be performed in the future.

In this study, we performed AFSCs transplantation at six weeks post CTx that the ovaries have been impaired seriously, when compared to the healthy control, to evaluate the therapeutic effect of AFSCs apply in POF. Our findings indicated that the transplanted AFSCs rescued the fertility by preventing atresia of the remaining follicle. Since the CTx induced follicular atresia peaked within the first week after CTx and the continued decrease in the primordial follicle pool, it is reasonable to expect an attenuated therapeutic effect with the delay in timing of transplantation. In the clinical applications, it might be favorable to administer the AFSCs closer to or even prior to CTx to better preserve follicle reserve from destruction and prevent POF from occurrence so that the maximal efficacy shall be achieved.

Collectively, our findings demonstrated that AFSCs may prevent follicle atresia in the ovaries of mice with POF and restore fertility. These findings suggest the potential of AFSCs to treat infertility.

## Supporting Information

Figure S1 **Appearance and histological analyses of ovaries of mice with CTx.** (A) The appearance of ovary between non-chemotherapy (CTx)-mouse (Con, left) and CTx-mouse (CTx, right) showed the severely atrophic ovary in CTx-mice followed by CTx for six weeks. Scale bar = 1 mm. (B) The weight of ovaries of CTx-mice compared with those of non-CTx-mice (Con) (n = 3). Data represent the mean ± standard error of mean (S.E.M.) *P<0.05. (C, D) Hematoxylin-eosin staining of ovaries of non-CTx-mice (C) and CTx-mice (D). Arrows indicate developing follicles. Scale bar = 100 µm.
(TIF)
