# Supplementary figures and images for: Amniotic Fluid Stem Cells Prevent Follicle Atresia and Rescue Fertility of Mice with Premature Ovarian Failure Induced by Chemotherapy
Source: PLoS One. 2014 Sep 8;9(9):e106538. doi: 10.1371/journal.pone.0106538 (PMC4157795; doi:10.1371/journal.pone.0106538)

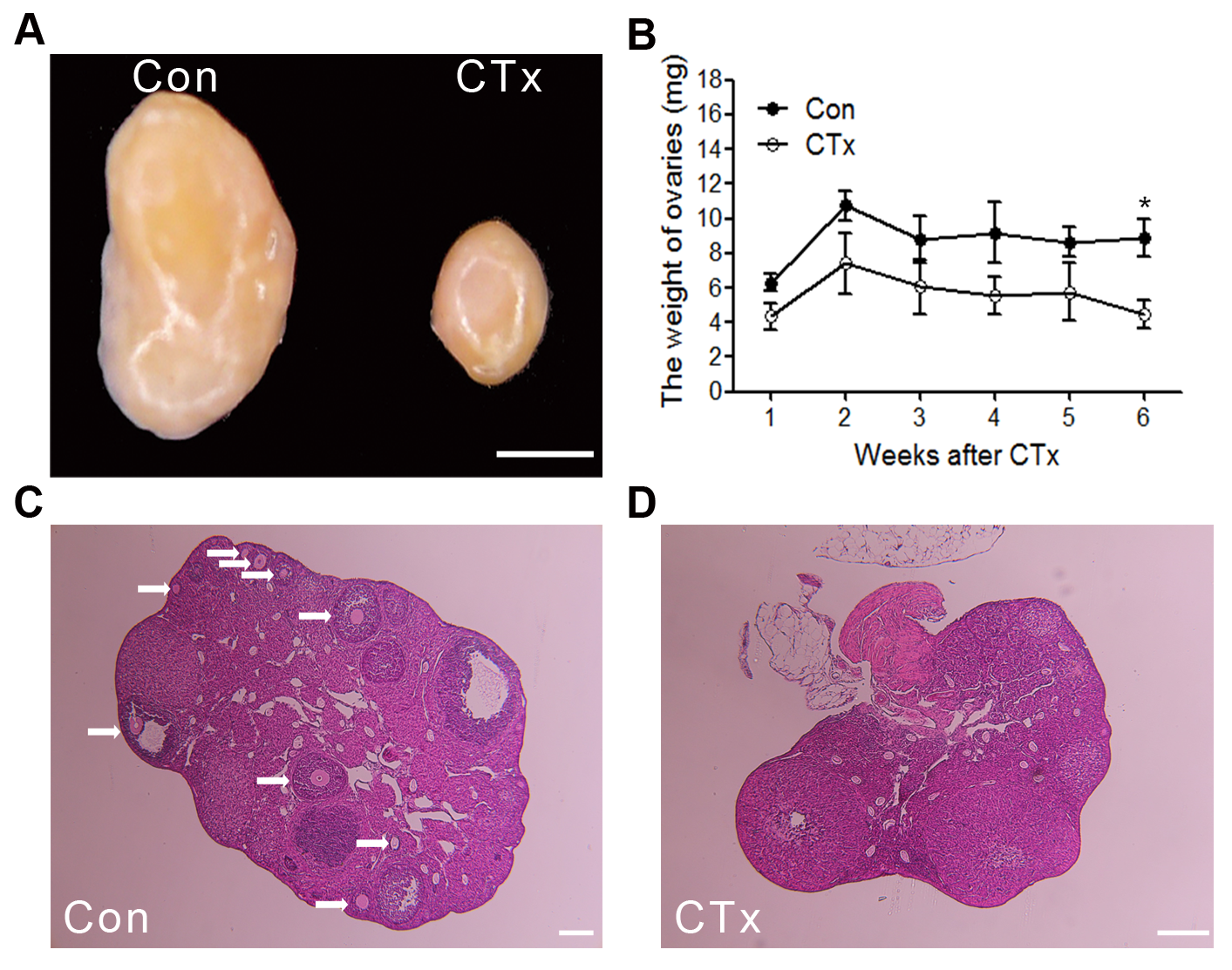

Supplement: Figure S1 — Appearance and histological analyses of ovaries of mice with CTx. (A) The appearance of ovary between non-chemotherapy (CTx)-mouse (Con, left) and CTx-mouse (CTx, right) showed the severely atrophic ovary in CTx-mice followed by CTx for six weeks. Scale bar = 1 mm. (B) The weight of ovaries of CTx-mice compared with those of non-CTx-mice (Con) (n = 3). Data represent the mean ± standard error of mean (S.E.M.) *P<0.05. (C, D) Hematoxylin-eosin staining of ovaries of non-CTx-mice (C) and CTx-mice (D). Arrows indicate developing follicles. Scale bar = 100 µm. (TIF) [file pone.0106538.s001.tif]
